# Clinical Impact of Electronic Monitoring Devices of Inhalers in Adults with Asthma or COPD: A Systematic Review and Meta-Analysis

**DOI:** 10.3390/ph16030414

**Published:** 2023-03-08

**Authors:** Noe Garin, Borja Zarate-Tamames, Laura Gras-Martin, Raimon Milà, Astrid Crespo-Lessmann, Elena Curto, Marta Hernandez, Conxita Mestres, Vicente Plaza

**Affiliations:** 1 Department of Pharmacy, Hospital de la Santa Creu i Sant Pau, Universitat Autònoma de Barcelona, 08025 Barcelona, Spain; 2 School of Health Science Blanquerna, Universitat Ramon Llull, 08025 Barcelona, Spain; 3 Centro de Investigación Biomédica en Red de Salud Mental (CIBERSAM), Instituto de Salud Carlos III, 28007 Madrid, Spain; 4 Department of Medicine, Universitat Autònoma de Barcelona, 08025 Barcelona, Spain; 5 Department of Respiratory Medicine, Hospital de la Santa Creu i Sant Pau, Universitat Autònoma de Barcelona, 08025 Barcelona, Spain

**Keywords:** asthma, COPD, meta-analysis, electronic monitoring device, medication adherence, clinical outcomes

## Abstract

We conducted a systematic review and meta-analysis to gain insight into the characteristics and clinical impact of electronic monitoring devices of inhalers (EMDs) and their clinical interventions in adult patients with asthma or COPD. The search included PubMed, Web of Science, Cochrane, Scopus and Embase databases, as well as official EMDs websites. We found eight observational studies and ten clinical trials, assessing a wide range of clinical outcomes. Results from the meta-analysis on adherence to inhalers in a period over three months were favourable in the EMD group (fixed effects model: SMD: 0.36 [0.25–0.48]; random effects model SMD: 0.41 [0.22–0.60]). An exploratory meta-analysis found an improvement in ACT score (fixed effect model SMD: 0.25 [0.11–0.39]; random effects model: SMD: 0.47 [−0.14–1.08]). Other clinical outcomes showed mixed results in the descriptive analyses. The findings of this review highlight the benefits of EMDs in the optimization of adherence to inhaled therapy as well as the potential interest in other clinical outcomes.

## 1. Introduction

Among chronic respiratory diseases, asthma and chronic obstructive pulmonary disease (COPD) stand out for their high prevalence, impact on quality of life and clinical repercussion. Both pathologies share some characteristics, such as airflow obstruction measured as forced expiratory volume in 1 s/forced vital capacity (FEV1/FVC) < 0.7, and the importance of inhaled treatment to achieve the best possible control, which will depend on the stage or severity of the patient, along with other strategies such as control of risk factors or smoking cessation. However, these pathologies are far from being controlled in multiple cases [1,2,3].

Adherence to pharmacological treatment is poor in chronic pathologies, close to 50% according to estimates by the World Health Organization [4]. Moreover, administration of inhaled therapy is correct in a third of the patients [5]. Likewise, adequate adherence to inhalers is associated with a reduction in asthma exacerbations, greater control of symptoms, lower systemic cortico-steroid requirements and lower disease-related mortality [6,7,8,9,10]. In COPD, several studies associate poorer adherence to inhalers with a higher number of exacerbations, admissions and mortality [11,12,13,14]. According to data from a Registry of Pharmaceutical provision in Spain, in 2017 only 63% of all prescribed prescriptions for inhalers (adrenergic receptor agonists, corticosteroids, anticholinergics and their combinations) were dispensed [15].

Despite technology advances in pulmonary delivery devices over the last years, adherence and their correct use remains a major issue in clinical practice [16]. Most of the adherence measures used in clinical practice rely on patients’ self-report data, including general questionnaires applied to respiratory diseases such as the Morisky Medication Adherence Scale [17,18] and specific questionnaires such as the Test of Adherence to Inhalers [19]. However, over-reporting from patients may be compromising the accuracy of these tools in numerous cases [20,21]. Objective measures, such is the case of the assessment of dispensing records, may overcome this barrier but face the possibility of the dispensed medication not being administered or connection problems in the pharmacy systems being present [15,22]. A novel approach to cope with these difficulties is the use of electronic monitoring devices (EMDs) attached to inhalers as an objective measure of adherence.

In this context, one of the most innovative proposals is the use of sensors attached to inhalers, in such a way that their use is electronically recorded and communicated to the patient, the clinical team or both [23]. With this information, interventions aimed to improve adherence can be conducted at any of those levels, allowing patient engagement and continuing education and the participation of the multidisciplinary team, with the possibility of including activities and interventions covering other clinical problems of interest. Several promising experiences have been published indicating its potential in improving adherence and other clinical parameters of interest. For example, its employment has been related to a reduction in the use of rescue inhalers, improved results of the Asthma Control Test (ACT), increased rescue inhaler-free days and increased adherence to inhalers [23,24,25,26,27,28]. There have been some interesting efforts to summarize the characteristics of EMDs but some of them focus on functionalities, while others are literature or scoping reviews targeting technological interventions in a broad sense or including different types of patients (e.g., paediatrics and adults) that mostly include data from conference abstracts [29,30,31,32,33,34,35] rather than complete, peer-reviewed articles. Thus, we aimed to explore the characteristics and assess the impact of EMDs and their clinical interventions in adult patients with asthma or COPD by means of a systematic review and meta-analysis.

## 2. Methods

### 2.1. Study Registration

This systematic review and meta-analysis were prospectively registered in the International Prospective Register of Systematic Reviews (PROSPERO), identification code CRD42022318919. The protocol adhered to the Preferred Reporting Items for Systematic Reviews and Meta-Analyses (PRISMA) guidelines [36].

### 2.2. Eligibility Criteria

A systematic review through computer searches was performed to identify studies on adult patients (≥18 years) with asthma and/or COPD that measured the clinical impact related to interventions based on EMDs. The outcomes assessed in the review were open to any clinical variable due to the potential broad range of interest in practice, such as: adherence, use of reliever medication, symptoms, exacerbations, Emergency Department (ED) visits, hospitalizations, pulmonary function tests and other relevant clinical outcomes. We included observational studies and clinical trials to gather all relevant information of the use of the EMDs and the nature of the interventions as this is a very novel approach, with the additional intention of conducting a meta-analysis with the results of randomized clinical trials. We included studies regardless of the nature of the comparator group in the case of randomized trials.

We included studies in the English and Spanish language, with no time restrictions. Unpublished articles and conference abstracts were excluded. In addition, studies were considered ineligible when the outcomes of interest were not measured or reported.

### 2.3. Information Sources

The search was conducted on 1 June 2022 on the following bibliographic databases: PubMed, Web of Science, Cochrane, Scopus and Embase, according to the eligibility criteria, with no time restrictions.

An additional search was performed including cites in reviews found in the previous search, citation examination of the selected articles and in the websites for Digihaler^®^ (www.digihalerhcp.com), HeroTracker^®^ (www.coherohealth.com), Respiro Amiko^®^ (www.amiko.io), CapMedic^®^ (www.capmedicinhaler.com), Turbuplus™ (www.turbuplusinfo.co.uk), Propeller Health^®^ (www.propellerhealth.com), INCA™ (www.incadevice.com) and Hailie^®^ (www.hailie.com) (35). Accessed on 1 August 2022.

### 2.4. Search Strategy

**Figure 1:**
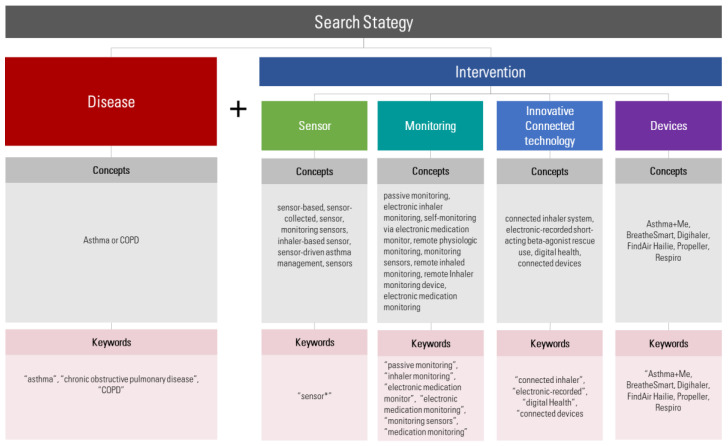
Search strategy summary.

Firstly, an exploratory phase was conducted to detect the terminology used for electronical interventions by means of reviewing original articles presented in some of the aforementioned websites. The following four domains were found and developed with their corresponding keywords: sensor, monitoring, innovative connected technology and devices (Figure 1). The full search strategy is available in File S1. The search review process was assessed with the Peer Review of Electronic Search Strategies (PRESS) checklist [37].

### 2.5. Selection and Data Collection Process

The data from searches in each database were exported to RefWorks, and an initial phase for detection of duplicates was performed. A subsequent duplication detection phase was conducted in the resulting database by DOI identifier and manual assessment. The results of the refined database were screened (title/abstract) by two independent investigators, with disagreements resolved by a third researcher. This method was replicated for the following full-text assessment and final inclusion of articles. Additionally, identification of studies via websites and cites in systematic reviews and included studies (see above) was performed by an independent researcher, with a subsequent validation by another researcher. Once selected, data were collected and validated by two independent investigators from each report.

### 2.6. Data Items and Synthesis Methods

The outcomes of the review were divided into domains and their corresponding items to be assessed:

- Adherence to chronic inhaled treatment.
- Control and symptoms of the disease. E.g., ACT, Asthma Control Questionnaire (ACQ), modified Medical Research Council (mMRC) scale, COPD assessment test (CAT), etc.
- Quality of life.
- Acute worsening and related issues (use of reliever inhaled treatment, exacerbations, visits to ED, hospital admissions, primary care and need of systemic corticosteroids).
- Pulmonary function tests results.
- Other relevant clinical outcomes.

All results compatible with each outcome domain were sought, regardless of the time frame of measurement. The data collected also included the possibility of “other” clinical variables as we were exploring a novel approach, and it was plausible that other relevant data may be available. Due to the nature of EMDs, we anticipated that adherence to medication may be the most frequent variable found in the studies.

The other variables collected were author, year of publication, sample size, age of participants, study design, number of centres, follow-up period, control group, intervention group, intervention details, recruitment setting, healthcare professional involved, circuit type (focused on the healthcare professional, the patient or both of them), type of interaction, type of inhaler assessed, type of EMD, data recorded and health outcomes.

### 2.7. Study and Report of Risk of Bias Assessment

The risk of bias was assessed with the Revised Cochrane risk-of-bias tool for randomized trials (RoB2) [38] and with the Methodological index for non-randomized studies (MINORS) [39]. All methodological components/items of the tools were applied. Assessment was performed by a researcher and validated by a second member of the team. Reporting of risk of bias was summarized in the text and in graphical figures for independent items and global results of each study.

### 2.8. Statistical Analysis

The descriptive data of the participants’ characteristics were reported as a mean (SD). All meta-analyses’ calculations were conducted with the R software (Vienna, Austria) with meta and metafor packages for meta-analysis (Version 3.5.1.). Descriptive analyses and figures of the risk of bias were performed using Microsoft Excel for MAC, version 16.29.1 (Microsoft, Redmond, WA, USA). The mean and standardized mean differences (Hedges’ g) and 95% CI for each group were calculated. The analysis of pooled data was conducted using a random-effect model to estimate the change for each group at the same measurement time on primary and secondary outcomes. Standardized mean differences were weighted by the inverse of the variance to calculate the size of the effect and 95% confidence interval. Cohen’s criteria were used to interpret the magnitude of the effect: <|0.50|: small; |0.50| to |0.80|: moderate; and >|0.80|: large. Heterogeneity was assessed using Cochran’s Q statistics and its corresponding *p*-value as well as the I2 statistic, which describes the percentage of variability in effect estimates attributable to heterogeneity rather than chance when I2 was >30% (30–60% representing moderate heterogeneity). Publication bias was assessed with funnel plots and Begg’s test. Significance was set at *p* < 0.05.

## 3. Results

### 3.1. Study Selection

**Figure 2:**
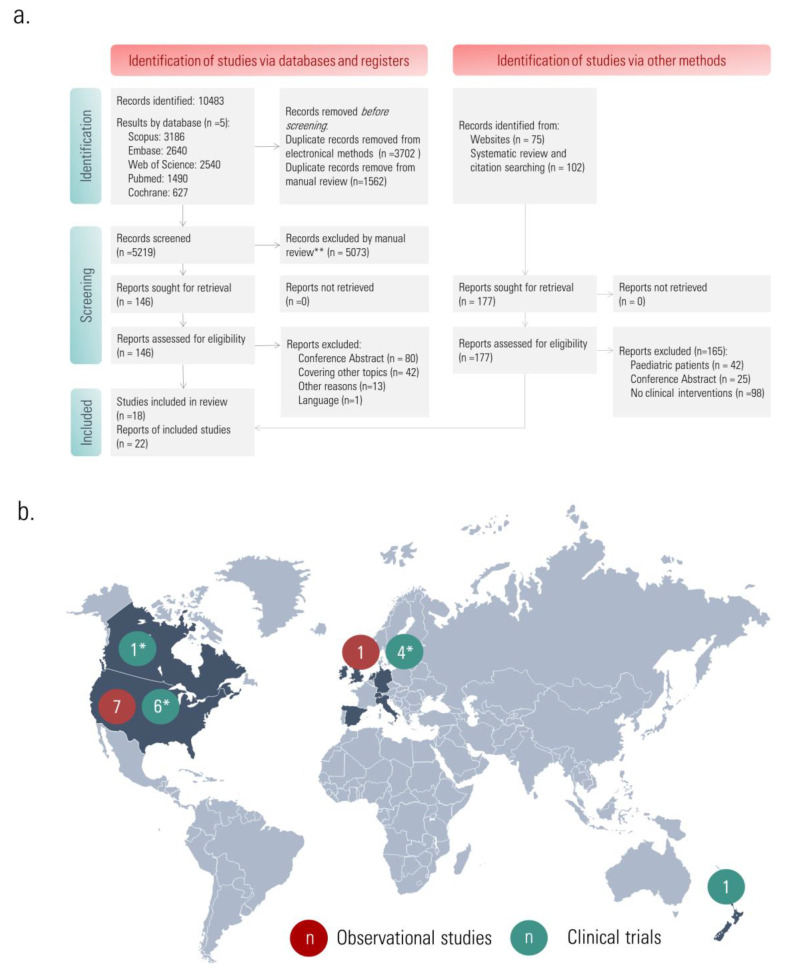
Search results. (**a**) PRISMA Flow Diagram; (**b**) distribution of studies by country. * One clinical trial was a multi-country study [51]: Canada, Germany, Italy, Netherlands, Spain, UK and the USA.

A total of 10483 articles were identified through computer searches in the selected databases, with 5264 duplicates removed through electronical or manual methods. A total of 5219 studies were screened by title and abstract and 146 were assessed for eligibility by full-text assessment. An additional search was performed using websites, and citation searching of articles and systematic reviews, with 177 articles assessed for eligibility. Some of the retrieved studies might appear to meet the inclusion criteria, but were eventually discarded due to several reasons, such as: not providing separate results from adults and paediatric patients [40,41], using EMDs to confirm the validity of the response to the Fractional Exhaled nitric oxide (FeNO) outpatient test, not as an intervention itself [42], or assessing patient satisfaction with EMDs rather than its clinical impact on disease as study outcomes [43]. Eighteen studies were finally included in the systematic review (Figure 2). Seven out of eight observational studies were conducted in the USA, while one study was conducted in the Netherlands. The distribution of clinical trials was the USA (n = 5), Ireland (n = 2), New Zealand (n = 1), Switzerland (n = 1) and one multi-country study (Canada, Germany, Italy, Netherlands, Spain, UK and the USA).

### 3.2. Study Characteristics and Results

**Figure 3:**
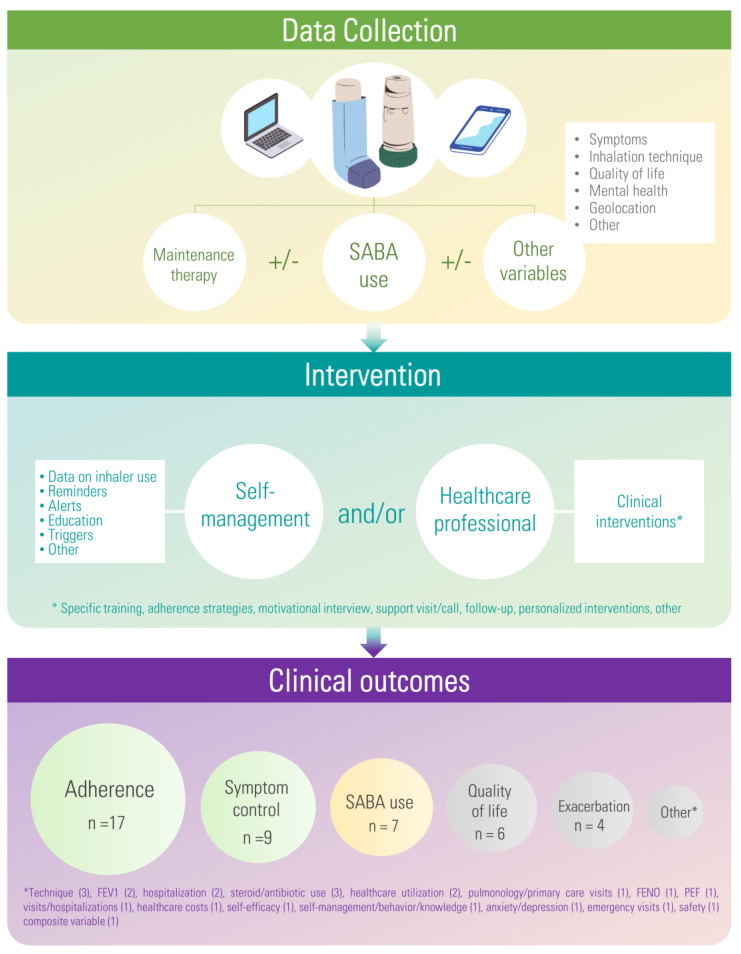
Visual summary of results. Colour guide in the clinical outcomes section: green (positive effects in some studies, confirmed in the meta–analysis); yellow (positive effects in some studies, meta–analysis not performed); and grey (mixed or no effects with the data available). Acronyms: n (number of studies assessing a specific outcome); SABA (short-acting beta agonist).

We found eight observational studies [28,44,45,46,47,48,49,50] and 10 clinical trials meeting the criteria [23,27,51,52,53,54,55,56,57,58,59,60,61,62] (Figure 3, Table 1 and Table 2).

Regarding observational studies, six of them focused on patients with COPD, one study on asthma and one study on patients with asthma and/or COPD (Table 1). Their sample size ranged from n = 11 to 2292 patients, with an average age ranging from 36.8 to 68.6 years and being over 60 years in five out of the eight studies. Clinical outcomes assessed in observational studies included adherence to maintenance medication, use of rescue medication, disease control, inhalation technique, quality of life, self-management variables (e.g., behaviour, knowledge and adherence to the intervention), steroid use, health-care utilization variables and mental health scores. Variables were differently operationalized across studies (e.g., mean daily short-acting beta-agonist [SABA] use, change in mean of SABA use, percentage of SABA-free days, etc.). Six of the studies performed statistical analyses of the clinical outcomes [28,44,46,47,49,50], while two studies presented descriptive analyses only [45,48]. Adherence to maintenance inhalers improved in two studies [49,50] whereas it was similar or decreased in the other two studies [44,46]. SABA use was found to be decreased in all variables assessed in two different studies [28,44]. One study focused on symptom control and found a clinical improvement in ACT score, days with asthma symptoms and nights with asthma symptoms. However, no difference was found regarding activity limitations [47]. Finally, one study found a decrease in COPD-related healthcare utilization and high accuracy of rescue alerts in predicting moderate–severe exacerbations but found no differences in variables such as all-cause healthcare utilization, number of pulmonary or primary care visits and antibiotic or steroid use [46].

As for the clinical trials, seven studies focused on asthma, one study on COPD and two studies on asthma and/or COPD (Table 1). Their sample size ranged from 19 in a pilot clinical trial to 437 participants, with an average age ranging from 36 to 70 years. Clinical outcomes assessed in clinical trials comprised adherence to maintenance medication, use of rescue medication, disease control, inhalation technique, quality of life, self-management variables, self-reported symptoms, exacerbations, lung function tests, hospitalizations, steroids use, ED visits and composite clinical variables. Variables were differently operationalized across studies resulting in great variability across studies (e.g., time to next exacerbation, exacerbation rate, severe exacerbations leading to hospitalization, etc.) (complete list of variables assessed in clinical trials: File S2). Adherence to maintenance inhalers was assessed in eight out of the ten clinical trials. The intervention group showed a statistically significant improvement in adherence compared with the control in six of the trials at the end of the study analysis [51,53,54,55,56,57,58,59,60,61]. Two studies found no differences in adherence results [27,52]. Regarding SABA use, mixed results were found in three studies, as improvement or no difference was found depending on the variable or subgroup assessed (e.g., SABA use vs. SABA-free days) [23,27,51]. No differences in SABA use were found in one study [61]. Studies assessing disease control with ACT or ACQ questionnaires showed mixed results: three studies found no differences [27,51,52], one study showed better results in the intervention group [62] and one study showed improvement for the previously uncontrolled subgroup only [23]. One study assessed independently additional symptoms (cough, breathlessness, nocturnal symptoms, wheeze, etc.). The intervention group improved all the symptoms over the study period, but no direct comparison across groups was performed [58]. In relation to quality of life, four trials found no differences between groups [52,55,60,62] and one study found better results over time in the intervention group [58]. As for other variables, none found statistical significance: FeNO, PEF, exacerbations, time to exacerbations, steroids use, ED visits, hospitalizations, FEV1, technique error, critical technique error rate and composite clinical variable criteria [27,51,52,53,54,55,56,57,58,59,60,61].

### 3.3. Monitoring Sensor-Based Interventions

Methodology of the interventions varied greatly across the studies. For example, recruitment and co-ordination settings included hospitals, medical clinics, community care, community pharmacies, universities, social media campaigns, online registries or was not specified. Healthcare professionals involved in the studies were not specified in eight studies, while they were varied across the studies providing this information: healthcare educator, nurse, pharmacist, physician (allergist, immunologist, pulmonologist, etc.) and physiotherapist. The most frequently used EMD was Propeller health^®^ (Madison, WI, USA), used in eight of the studies. Other studies based their monitoring in Chronologs (Medtrac Technologies, Model MC-311, Lakewood, CO, USA); Diskus Adherence Logger (DAL, developed by a study researcher); INCATM (Inhaler Compliance AssessmentTM); MDILog^®^ (Life Link Monitoring, Inc, Kingston, NY, USA); Nebulizer Chronolog (Forefront Engineering Corp, Denver, Colo); POEMS (Pharmis GmbH, Beinwil am See, Switzerland); Smartinhaler (Adherium Ltd., Auckback, New Zealand); SmartTrack^®^ device (Nexus6, Auckland, New Zealand); and Respiro^®^ (Amiko Digital Health Limited, London, UK). Sensors were attached to pMDI in seven studies [23,28,44,47,50,61,62], to DPI in three studies [45,53,58], both to pMDI and DPI in four studies [48,51,52,55] and was not specified in four studies [27,46,49,60]. Data from EMDs lead to interactions with patients through smartphone applications in eight studies [23,27,28,44,48,49,51,55]. Other means of interconnectivity with patients or healthcare professionals were web dashboards, e-health application modules on tablets or PCs, online interfaces/web browsers, web applications, platform-generated emails, robot devices, phone calls or face-to-face sessions. Some smartphone applications and additional means of interconnectivity allowed patients to provide, autonomously, data on variables such as ACT, symptoms, self-efficacy or QOL in several studies. Feedback and related actions from the monitoring sensor system were patient-oriented in five studies [44,45,47,48,49], healthcare professional-oriented in five studies [46,52,53,58,61] and comprised both patients and healthcare professionals in eight studies [23,27,28,50,51,55,60,62]. Patients received self-management interventions in 13 studies, with a varied range of actions: feedback of inhaler use, reminders, automated decision support system, gamified challenges, identification of potential triggers and guidelines-based education [23,27,28,44,45,47,48,49,51,52,53,55,60]. In the studies including healthcare professionals, interventions included medication adjustments, phone follow-up, problem-solving assistance, open-ended conversations, face-to-face reviews, e-mail advice, support calls, provision of individual behavioural strategies, general education sessions, visual feedback and decision-making tool interventions [23,27,28,46,50,51,52,53,55,58,60,61,62].

### 3.4. Risk of Bias in Studies

**Figure 4:**
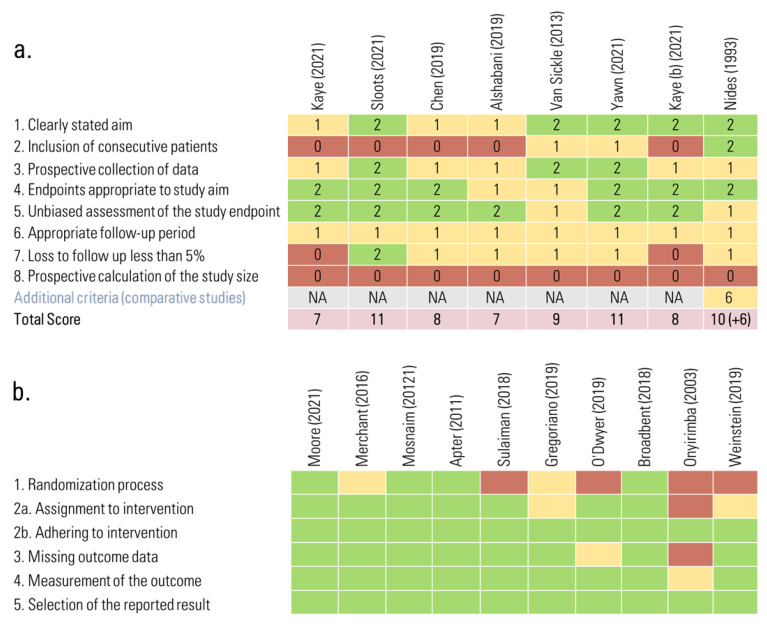
Risk-of-bias assessment. (**a**) Observational studies; (**b**) clinical trials. *NA: not applicable.* References: [23,27,28,44,45,46,47,48,49,50,51,52,53,55,58,60,61,62].

A separate assessment of risk of bias was conducted for observational and clinical trials (Figure 4). Regarding observational studies, we obtained a range from 7 to 11 points with the MINORS tool (0 = lowest risk of bias; 16: higher risk of bias) [28,44,45,46,47,48,49,50]. One observational study scored 16 points in the additional assessment of comparative projects (0 = lowest risk of bias; 24: higher risk of bias) [50]. As for the clinical trials, the assessment was performed with the RoB2 tool. Five studies were assessed as having low risk-of-bias criteria [23,27,51,52,60], four studies as high risk-of-bias criteria [53,58,61,62] and one study was classified as having some concerns criteria [55]. For studies with high risk of bias, the main reason was potential bias arising from the randomization process criteria due to the inherent use of electronic devices and consequent interventions [53,58,61,62]. Due to the nature and small number of studies, no exclusions for this reason were pre-established in the meta-analysis.

### 3.5. Impact of Interventions on Adherence to Maintenance Inhaled Therapy

A total of six trials were found to measure adherence in percentage as a clinical outcome.

**Figure 5:**
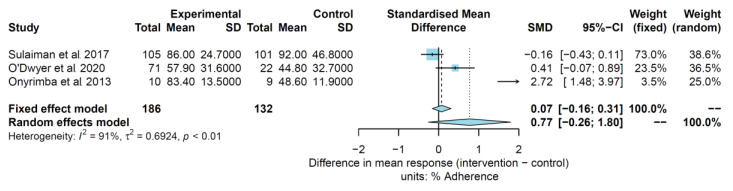
Adherence outcomes up to 3 months. References: [53,58,61].

The impact of interventions in studies assessing adherence up to 3 months was assessed in three studies, including 318 participants [53,58,61]. Figure 5 shows the results of the forest plot analysis. Although adherence results were favourable in the intervention group, no significant differences were found in both the fixed effect model analysis (SMD: 0.07 [−0.16 to 0.31]] or the random effects model analysis (SMD: 0.77 [−0.26 to 1.80]]. The estimate of the between-study variance (heterogeneity) was considered high (I^2^ = 91%; τ^2^ = 0.69; *p* = 0.01).

**Figure 6:**
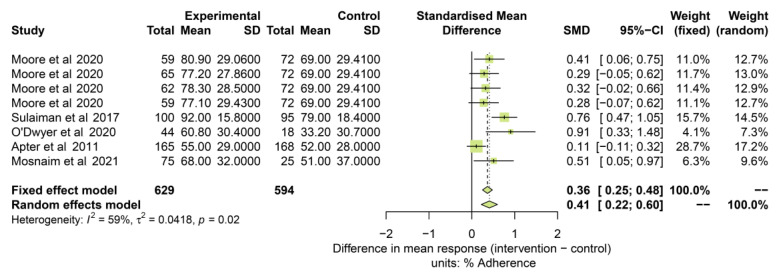
Adherence outcomes in studies 3 months or longer. References: [27,51,52,53,58].

The impact of interventions in studies assessing adherence in a period of 3 months or longer was assessed in five studies, including 1223 participants [27,51,52,53,58]. Figure 6 shows the results of the forest plot analysis. Adherence results were favourable in the intervention group in all studies and resulted in significant differences for both the fixed effect model analysis (SMD: 0.36 [0.25 to 0.48]) or the random effects model analysis (SMD: 0.41 [0.22 to 0.60]). The estimate of the between-study variance (heterogeneity) was considered moderate (I2 = 59%; τ^2^ = 0.04; *p* = 0.02).

### 3.6. Impact of Interventions on Other Clinical Outcomes

**Figure 7:**
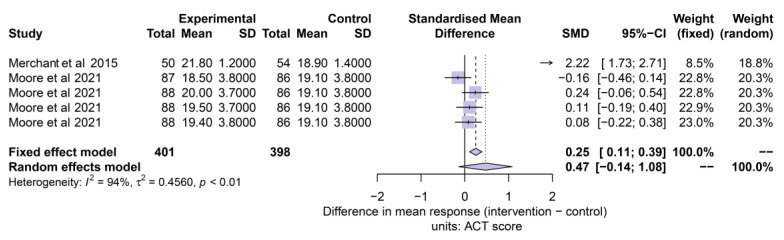
Asthma control test (ACT) outcomes. References: [23,51].

The variability of other variables in terms of definition, operationalization and study timeline duration prevented us to perform additional meta-analysis. However, two of the trials were found to measure control with the ACT questionnaire as a clinical outcome, leading to an exploratory assessment with 799 individuals [23,51]. Figure 7 shows the results of the forest plot analysis. ACT results were favourable in the intervention group. Significant differences were found in the fixed effect model analysis (SMD: 0.25 [0.11 to 0.39]] but not in the random effects model analysis (SMD: 0.47 [−0.14 to 1.08]]. The estimate of the between-study variance (heterogeneity) was considered high (I^2^ = 94%; τ^2^ = 0.46; *p* < 0.01).

## 4. Discussion

To the best of our knowledge, this is the first systematic review with meta-analysis exploring the characteristics and assessing the impact of clinical interventions derived from data of EMDs in adult patients with asthma or COPD. The most remarkable result to emerge from our study is the positive impact of EMD-based interventional programmes on adherence to inhaler treatment and the tendency to positive or mixed results in other outcomes such as symptom control, with potential benefits for daily practice in the optimization of inhaled therapy management.

Firstly, we would like to highlight the impact of these interventions on adherence. Adherence on maintenance therapy was the most frequent clinical outcome assessed in both the observational studies and clinical trials. Mixed-favourable results were found in the observational studies, which principally focused on COPD patients. However, the clinical trials showed an improvement in adherence for the intervention group in the meta-analysis. Although most trials focused on asthma patients, those studies focusing exclusively on COPD patients or including both participants with COPD or asthma also showed an improvement in adherence, which suggests that COPD patients may also benefit from this type of interventions. Poor adherence is an acknowledged risk factor by GINA and GOLD guidelines to symptom burdens, exacerbations, and poor quality of life to be assessed and tackled by the multidisciplinary team [1,63]. Inhaler treatment involves factors contributing to suboptimal adherence at three levels: medication, unintentional and intentional issues [64]. One of the main relevant results of the review is the ability of these interventional programmes to include a wide range of aspects related to these factors, such as patient self-awareness and efficacy, disease and management education, inhaler technique, triggers, age-related factors such as comorbidities, misunderstood directions, forgetfulness, dissatisfaction or inappropriate expectations [64]. The methodology of interventions tackling adherence in the assessed studies was aligned with successful interventions to improve adherence in previous studies regardless of the use of monitoring devices, such as shared decision making, electronic reminders, visits and providing information to clinicians [65,66,67,68,69,70,71].

As for the rest of the variables, mixed results were found for SABA use and symptoms of asthma and COPD. Theoretically, positive results may be obtained if adherence is improved but these clinical outcomes could be shaped by additional factors such as weather, infections, environmental triggers, comorbidities, severity of illness and other factors. These aspects may have a higher impact on other clinical outcomes and be responsible for the lack of results in variables such as exacerbations, hospitalizations or spirometry results. However, some studies on children have found fewer exacerbations requiring oral corticosteroids at 12 months [67]. With this in mind, adherence management would be the primary goal of EMDs due to the nature of the data collected by the sensors, but there is a need for further research to assess their real clinical benefits in terms of other health outcomes or to what extent it would be useful to implement specific additional interventions linked to these programmes as a global strategy [35].

A high degree of variability is inherent in the development of new technologies. In our review, we found 10 different EMDs, in line with previous literature [29,30,31,32,33,34,35]. Technological differences between their inhaler sensors should not pose a differential problem as they are validated systems. However, the associated digital-engagement tools, dashboards, data available, clinical setting, specific professionals involved, the number of associated interventions, usability and acceptability could make a difference between them [29,30,31,32,33,34,35,72]. In our review we have found a great variability in terms of clinical setting, professionals involved, follow-up, interventions, availability of smartphone applications and whether the clinical approach was focused on the patient, the healthcare professional or both. To what extent these factors may impact the clinical results remain uncertain. However, the results of our review show that all clinical trials included healthcare professionals as key roles in the intervention, highlighting the importance of interventions conducted by healthcare professionals rather than focusing on self-management with apps only [23,27,51,52,53,54,55,56,57,58,59,60,61,62] (Table 2).

Cost-effectiveness and feasibility are essential concerns to be considered when translating interventional programmes based on data from monitoring devices into regular clinical practice. On one hand, there are direct costs associated with monitoring devices, e-health platforms, and related-products and interventions. Minimizing the cost of monitoring devices has been described as a relevant acceptability criterion to assess device characteristics [73]. On the other hand, suboptimal inhaler use results in poor clinical outcomes, with studies reporting increased direct and indirect costs that may be potentially reduced [74,75]. In this context, an economic analysis of monitoring devices and adherence-related interventions showed it may be cost-effective and cost-saving [76]. In terms of feasibility, some inexperienced patients face a considerable number of challenges, such as sensory impairment, intellectual ability, motivation, reduction of fine motor control, low self-efficacy of technology, fear or dislike of electronic devices, inexperience with e-health or computers, lack of awareness of e-health opportunities, previous unmet expectations, fear or losing traditional health services or lack of smart phones [77]. With regard to healthcare professionals, apart from e-health literacy skills, there is a need of training on EMD functioning, checking alerts, dashboards and typical errors [78]. Healthcare professionals have highlighted a high degree of administrative burden and complexity of these interventions, that would require additional employees to handle the corresponding workload [79]. Furthermore, a study showed that providing adherence information to healthcare professionals did not improve adherence unless the professional deliberately decided to check the details of a specific patient [69]. Moreover, the existence of an increasing number of EMDs can complicate their implementation and management in regular practice. Thus, a careful selection of patients who are most likely to benefit from interventions and the development of a common framework of the platforms seem to be convenient options to be prioritized in the near future.

Our systematic review and meta-analysis have some limitations. Firstly, we found a great variability of clinical outcomes, operationalization of variables and follow-up length periods across studies, which could impact the results, their interpretation and comparability. In relation to the use of monitoring devices, patients in both the intervention and control groups can suffer from the Hawthorne Effect, which implies a change in behaviour since they know they are being monitored [80]. Another aspect to be considered is the decrease of user engagement to e-health interventions with time, its clinical impact and potential measures to minimize this effect [81,82]. In addition, some studies focused on severe cases while others focused on patients regardless of their severity, which could affect the results if specific subgroups benefited more from the intervention. Moreover, data on the use of biological agents in asthma were not provided and may represent a major cofounding factor. Furthermore, information on pharmacological-inhaled treatment and posology was not present in some studies. Moreover, some of the studies were pilot projects and may benefit from escalation and ulterior methodology improvements of this type of studies. With regard to the review process, some limitations may also arise. For example, innovative technologies do not count with a definite terminology, so some articles might not have been found in the title/abstract search if alternative nomenclature was used by some authors. This limitation may be minimized if we performed preliminary searches to find synonyms in potential studies. In addition, as this is a novel technology, it is possible that additional features and interventions may improve clinical results in future trials. Finally, data from real-life studies would be of interest.

The findings of our systematic review and meta-analysis confirm the positive impact of EMD-based interventions on adherence to inhalers in adults with asthma or COPD. Our data indicate that other clinical outcomes, such as symptom control, may also improve when using EMD interventional programmes, but further research is needed to confirm whether additional interventions would be necessary, as asthma and COPD clinical results also rely on additional aspects such as environmental issues, comorbidities, etc. The broad implication of the present research is that EMDs represent a valuable asset that should help healthcare providers to implement policies to address asthma and COPD management.

## Figures and Tables

**Table 1:** Summary of results. ACQ (Asthma Control Questionnaire); ACT (Asthma Control Test); AQLQ (Asthma Quality of Life Questionnaire); Asthma-related quality of life (AQOL); AE (asthma education); C (Control group); CAT (COPD assessment test); CI (confidence interval); CCQ (Clinical COPD questionnaire); COPD (chronic obstructive pulmonary disease); DPI (dry-powder inhaler); ED (Emergency Department); FeNO (Fractional Exhaled nitric oxide); FEV1 (forced expiratory volume in 1 s); HCP (healthcare professionals); I (intervention group); ICS (inhaled corticosteroid); NA (not available); ND (no difference); PEF (peak expiratory flow); pMDI (pressurised Metered Dose Inhaler); POEMS (Polymedication Electronic Monitoring System); SABA (short acting beta agonist); SGRQ (St. George’s Respiratory Questionnaire).

Author (Year)	Disease	Sample Size	Age	Study Design	Number of Centers	Primary Clinical Outcome	Secondary Clinical Outcome	Follow-Up	Control Group	Intervention Group	Device	Outcomes
Alshabani (2020) [46]	COPD	39	68.6 (9.9)	Observational (pilot study)	Unicentric	All-cause healthcare utilization	- COPD-related healthcare utilization - Number of pulmonary and/or primary care clinic visits - Adherence - Adherence determinants - Outpatient antibiotic and/or steroid courses - Adherence and healthcare utilization correlation - Accuracy of rescue alerts in predicting moderate-to-severe exacerbations	12 months	No	Access to information and related interventions by HCP.	Propeller Health^®^	- All-cause healthcare utilization: ND (decrease in the subgroup with audio-visual reminders) - COPD-related healthcare utilization: baseline: 2.2 (2.3) vs. follow-up: 3.4 (3.2) *p* < 0.01 - Number of pulmonary and/or primary care clinic visits: ND - Antibiotic and/or steroid courses: ND - Adherence: −0.46%/week *p* < 0.0001 - Adherence determinants: not found - Adherence and healthcare utilization correlation: not found - Accuracy of rescue alerts in predicting moderate-to-severe exacerbations: 27.9% (sensitivity); 99% (specificity)
Apter (2011) [52]	Asthma	333	49 (14)	Clinical trial (randomized controlled trial)	Multi-centric	- Adherence to ICS - AQOL - ACQ - FEV1 - ED (asthma) - ED (any cause) - Hospitalizations (asthma) - Hospitalizations (any cause)	None	6 months	Standard AE: sessions not related to the topics in the intervention group sessions.	Access to information and related interventions for HCP + Standard AE sessions.	Depending on the inhaler: - Diskus Adherence Logger - MDILog^®^	- Adherence to ICS: ND - AQOL: ND - ACQ: ND - FEV1: ND - ED for asthma: ND - ED for any cause: ND - Hospitalizations for asthma: ND - Hospitalizations for any cause: ND
Broadbent (2018) [60]	COPD	60	C: 69.10 (9.85) I: 70.57 (10.34)	Clinical trial (pilot randomized control trial)	Unicentric	Hospitalizations	- Adherence to medication - Quality of life - Healthcare costs	4 months	Routine care	Self-management (reminders, education, trends on adherence) for patients. Access to information and related interventions for HCP	Smartinhaler + iRobi robot	- Number of hospitalizations: ND - Adherence (%). ◦ control: 29.5 ◦ Intervention: 48.5 (*p* = 0.03; *p* = 0.07 after controlling for other variables) - Quality of life (CCQ): ND - Healthcare costs: ND
Chen (2019) [28]	COPD	190	68.0 (9.2)	Observational (pilot study)	Multi-centric	- Change in mean SABA use - Percentage of SABA-free days - Night-time SABA use	None	12 months	No	Self-management for patients. Access to information and related interventions by HCP.	Propeller Health^®^	- Change in mean SABA use: −1.9 (95% CI 1.7–2.1) - Percentage of SABA-free days: +36% (95% CI 33–39) - Night-time SABA use: −0.8 (95% CI −0.9–0.7)
Gregoriano (2019) [55]	Asthma, COPD	169	C: 69.0 (8.8) I: 64.7 (12.4)	Clinical trial (randomized controlled trial)	Multi-centric	Time to next exacerbation	- Exacerbation frequency-Severe exacerbation leading to hospitalization - Health-related quality of life - Adherence (several variables)	6 months	Routine care	Access to information and related interventions sessions for HCP. Also, daily reminders of maintenance inhalers for patients.	Depending on the inhaler: (a) Smartinhaler (b) POEMS	- Time to next exacerbation: ND - Frequency of exacerbations: ND - QOL: ND - Taking adherence (mean days in range, %) ▪ pMDI ◦ C: 60.1 ◦ I: 81.6 (*p* < 0.001) ▪ DPI ◦ C: 80.2 ◦ I: 89.6 (*p* = 0.01) - Timing adherence (% of correct dosing intervals during 24 h) ▪ pMDI ◦ C: 50.6 ◦ I: 68.9 (*p* < 0.01) ▪ DPI: ND - Gaps (% of days without inhalation) ▪ pMDI ◦ C: 11.7 ◦ I: 3.2 (*p* = 0.008) ▪ DPI ◦ C: 9.8 ◦ I: 4.6 (*p* = 0.009) - Maximal gap length ▪ pMDI ◦ C: 11.6 ◦ I: 1.6 (*p* = 0.025) ▪ DPI ◦ C: 5.9 (5.2) ◦ I: 2.6 (*p* = 0.002)
Kaye (2021) [44]	COPD	611	62 (8)	Observational	Not specified	- CAT score - Mean daily SABA use - Daily maintenance adherence	None	6 monhs	No	Self-management for patients	Propeller Health^®^	- CAT score: −0.9 (95% CI: −1.4–−0.4); *p* < 0.001 - Mean daily SABA use: −0.6 (95% CI −0.8–0.4) *p*< 0.001 - Daily maintenance adherence: −4 (95% CI −6.9; −1.2) *p* = 0.001
Kaye (2021) [49]	asthma, COPD	Asthma: 1629; COPD: 663	Asthma 39.4 (12.6) COPD 60.9 (8.3)	Observational	Multi-centric	- Maintenance medication adherence	None	8–97 Days	No	Self-management for patients	Propeller Health^®^	- Adherence (asthma): OR 2.08 (95% CI 1.98–2.19) < 0.001 - Adherence (COPD): 1.61 (95% CI 1.49–1.75) < 0.001
Merchant (2016) [23]	Asthma	345	36.0 (NA)	Clinical trial (randomized in parallel arms)	Multi-centric	SABA use	- ACT - Control of asthma	12 meses	Routine care	Self-management for patients. Access to information and related interventions by HCP.	Propeller Health^®^	Initially uncontrolled patients: - SABA use ◦ C: −0.51 ◦ I: −0.62 (*p* < 0.001) - SABA free days: ND - ACT score (change): ◦ C: +4.6 ◦ I: +6.2 (*p* = 0.009) - Asthma control (% ACT > 19) ◦ C: +49% ◦ I: +63% (*p* = 0.02) Initially controlled patients: - SABA use ◦ C: −0.12 ◦ I: −0.16 *p* = 0.001 - SABA free days: ND - ACT score (change): ND - Asthma control (% ACT > 19): ND
Moore (2021) [51]	Asthma	437	47 (15)	Clinical trial (Open-label, randomised, parallel group study)	Multi-centric	Adherence to maintenance therapy	- Rescue Free Days - ACT - FeNO - PEF - Safety	6 months	Routine care	Arm 1 (maintenance data to patients and HCPs) Arm 2 (maintenance data to patients only) Arm 3 (maintenance and rescue data to patients and HCPs) Arm 4 (maintenance and rescue data to patients only)	Propeller Health^®^	- Mean daily adherence in %: ◦ Arm 1: 80.9 (3.19) *p* <0.001 ◦ Arm 2: 77.2 (3.04) *p* = 0.016 ◦ Arm 3: 78.3 (3.11) *p* = 0.006 ◦ Arm 4: 77.1 (3.25) *p* = 0.018 ◦ Control: 69.0 (3.19) (REF) (b) Mean monthly % of rescue-free days: ◦ Arm 1: 81.1 (2.82) *p* = 0.118 ◦ Arm 2: 81.2 (2.66) *p* = 0.105 ◦ Arm 3: 85.6 (2.76) *p* = 0.002 ◦ Arm 4: 83.7 (2.80) *p* = 0.015 ◦ Arm 5: 76.4 (2.82) (REF) - ACT: ND - FeNO: ND - PEF: ND - Safety: descriptive data
Mosnaim (2021) [27]	Asthma	100	48.5 (12.3)	Clinical trial (randomized Controlled Trial)	Unicentric	SABA-free days	- Mean ICS adherence - Asthma control (ACT) - −10% increase SABA-free days - ACT improvement ≥3 points - ACT score from <20 to ≥20 - Asthma exacerbations - >1 course of oral corticosteroids - >1 ED visit or hospitalization	3 months	Routine care	Self-management for patients. Access to information, related interventions and periodical review with patients for HCP.	Propeller Health^®^	- SABA-free days (%) change over time: ◦ C: 6% control (*p* = 0.18). ◦ I: 19% (*p*< 0.001) - Mean ICS adherence (%) change over time. ◦ C: −17% (*p* < 0.01) ◦ I: +2% (*p* = 0.40) - Asthma control: ND - 10% increase SABA-free days: ND - ACT improvement ≥3 points: ND - ACT score from <20 to ≥20: ND - Asthma exacerbations: ND - >1 course of oral corticosteroids: ND - >1 ED visit or hospitalization: ND
Nides (1993) [50]	COPD	205 (I:116; C: 89)	I: 49.0 (6.4) C: 50.3 (6.3)	Observational (ancillary study of a clinical trial)	Multi-centric	Adherence to inhaler treatment	None	4 months	No	Self-management for patients. Access to information and related interventions by HCP.	Nebulizer Chronolog	Mean percent adherent days: feedback group: 60.2 (25.9) vs. 40.4 (28.2) *p*< 0.0001 NOTE: additional secondary variables of adherence were provided
O’Dwyer (2020) [58]	Asthma, COPD	152	I: 54 (15) C: 55 (13) C2: 53 (15)	Clinical trial (cluster randomized clinical trial)	Multi-centric	Adherence (%)	- Proportion with actual adherence ≥80% (%) - Proportion with actual adherence <50% (%) - Attempted adherence rate (%): - Critical technique error rate - QOL (SGRQ score) - Self-reported symptoms ◦ cough ◦ breathlessness ◦ nocturnal symptoms ◦ wheeze - Exacerbation rate	6 months	- Routine care - Other comparator: inhaler technique education	Access to information and related interventions for HCP.	INCA^TM^	- Adherence (%): ◦ Biofeedback group 60.8 vs. demonstration group 44.2 vs. control group 33.2. ◦ Biofeedback-demonstration: *p* = 0.025; Biofeedback-control: *p* = 0.004 (b) Proportion with actual adherence ≥80% (%): ◦ Biofeedback group 29.27 vs. demonstration group 14.29 vs. control group 1.00. Between group difference: 0.030. ◦ Biofeedback-demonstration: no difference; Biofeedback-control: *p* = 0.015 - Proportion with actual adherence <50% (%) ◦ Biofeedback group 31.7 vs. demonstration group 60.7 vs. control group 62.50. Between group difference: 0.023. ◦ Biofeedback-demonstration: *p* = 0.017; Biofeedback-control: *p* = 0.033 - Attempted adherence rate (%): ◦ Biofeedback group 77.7 vs. demonstration group 66.1 vs. control group 52.1. Between group difference: 0.027. ◦ Biofeedback-demonstration: *p* = 0.036 Biofeedback-control: *p* = 0.04 - Critical technique error rate ◦ Biofeedback group 17.5 vs. demonstration group 21.9 vs. control group 36.1. Between group difference: ND ◦ Biofeedback-demonstration: ND; Biofeedback-control: *p* = 0.032 - QOL (SGRQ score) ◦ Biofeedback group: −6.1 (*p* = 0.04) ◦ Other arms: ND - Self-reported symptoms (Magnitude of the results graphically described). Statistical value: ◦ cough: Biofeedback (*p* < 0.05); demonstration: ND ◦ breathlessness: biofeedback (*p* < 0.01); demonstration (*p* < 0.05) ◦ nocturnal symptoms: biofeedback (*p* < 0.05); demonstration: ND ◦ wheeze: Biofeedback (*p* < 0.05); demonstration (*p* < 0.05) - Exacerbation rate: ND
Onyirimba (2003) [61]	Asthma	19	I: 45 (11) C: 53 (14)	Clinical trial (randomized trial)	Unicentric	Adherence to maintenance therapy	- SABA use - Quality of life - FEV1	10 weeks	Asthma education and management plan	Access to information and related interventions for HCP + same intervention of control group	Chronologs	- Adherence (%): graphical data (*p* = 0.003) - SABA use: ND - Quality of life (AQLQ): ND - FEV1: ND
Sloots (2021) [45]	COPD	11	66.8 (2.9)	Observational (pilot study)	Multi-centric	Adherence to the e-health self-management intervention: - completing digital daily symptom diaries - following the advised actions - using inhaled medication	- Inhalation technique - Health-related quality of life - COPD self-management behavior and knowledge - COPD self-efficacy - Anxiety/and depression score	4 months	No	Self-management for patients	Respiro^®^	Descriptive analyses only (no comparison with baseline)
Sulaiman (2018) [53]	Asthma	218	49.2 (16.5)	Clinical trial (Randomised, controlled, open-label clinical trial)	Multi-centric	Adherence (critical errors with missed doses were combined in this variable)	- Average adherence from dose counter - Attempted rate - Overdoses - Missed doses: - Technique error rate - Composite variable including PEF, ACT, AQLQ and adherence	3 months	Intensive education: repeated training in inhaler use, adherence and disease management.	Access to information and related-interventions sessions for HCP + Standard Intensive education sessions.	INCA^TM^	- Adherence: ◦ Intensive education group: 63% ◦ Biofeedback group: 73% (*p* < 0.01) - Average adherence from dose counter: ND - Attempted rate ◦ Intensive education group: 82% ◦ Biofeedback group: 73% (*p* = 0.01) - Overdoses ◦ Intensive education group: 6 ◦ Biofeedback group: 3 (*p* = 0.02) - Missed doses: ND - Technique error rate: ND - Composite variable: ND
Van Sickle (2013) [47]	Asthma	29	36.8 (19–74)	Observational (pilot study)	Multi-centric	No clinical outcome	- ACT score - Activity limitations - Days with asthma symptoms - Nights with asthma symptoms	4 months	No	Self-management for patients. Access to information and related interventions by HCP.	Not specified	- ACT score: baseline: 17.6 (3.35) vs. follow-up: 20.1 (3.66) - Activity limitations: ND - Days with asthma symptoms: baseline: 4.84 (4,13) vs. follow-up: 2.77 (3.56) - Nights with asthma symptoms: baseline: 2.03 (3.35) vs. follow-up: 0.55 (0.98)
Weinstein (2019) [62]	Asthma	39	40 (23-69)	Clinical trial (pilot randomized control trial)	Unicentric	Asthma control (ACQ)	Adherence to medication (>60%)	3 months	Standard care	Self-management for patients. Training and access to information and related interventions for HCP.	SmartTrack device	- ACQ (change): ◦ C: +1.41 ◦ I: +1.11 (*p* = 0.046) - Adherence: descriptive data only
Yawn (2021) [48]	COPD	122	65.2 (8.6)	Observational (pilot study)	Multi-centric	No clinical outcome	- Adherence to maintenance medication - Rescue inhaler use - CAT scores - Modified COPD treatment ratio (mCTR)	24 weeks	No	Self-management for patients.	Propeller Health^®^	Descriptive analyses only (no comparison with baseline)

**Table 2:** Summary of methodology and interventions by studies. ACT (Asthma Control Test); AE (asthma education); CAT (COPD assessment test); COPD (chronic obstructive pulmonary disease); DPI (dry-powder inhaler); HADS (Hospital Anxiety and Depression Scale); HCP (healthcare professionals); ICS (inhaled corticosteroid); pMDI (pressurised Metered Dose Inhaler); POEMS (Polymedication Electronic Monitoring System); SABA (short acting beta agonist); SGRQ (St. George’s Respiratory Questionnaire).

Author (Year)	Disease	Setting	HCP Involved	Device	Type of Inhaler	Data Collected with the Monitoring Device and Related E-Health Systems	Follow-Up	Circuit Type	Type of Interaction	Intervention
Alshabani (2020) [46]	COPD	Medical Clinic	Not specified	Propeller Health^®^	Not specified	- SABA use - Maintenance medication use	12 months	HCP	- HCP: e-mail (platform generated alerts) - HCP + patient: open-ended conversations (unspecified means of communication)	- HCP: platform-generated alerts were emailed to the clinicians when maintenance therapy was not used for four consecutive days or when rescue inhaler use increased for a day by 1.64 times the standard deviation above their average. - Patient + HCP: patients were contacted by HCP and open-ended conversations were held aiming to foster adherence and detect exacerbations.
Apter (2011) [52]	Asthma	Primary care and asthma specialty practices in low-income neighbourhoods with a high prevalence of asthma	Not specified	Depending on the inhaler: - Diskus Adherence Logger - MDILog^®^	pMDI, DPI	- Maintenance ICS use	6 months	HCP	- HCP: computer dashboard - Face-to-face sessions	- HCP: access to data from inhaler use to develop interventions. - Patient + HCP: Four 30-min sessions related to self-management, adherence or ICS therapy involving discussion of problem-solving, adherence or related issues. Data of inhalers use were showed to patients in these sessions. Furthermore, the same Standard AE sessions as in the control group were performed.
Broadbent (2018) [60]	COPD	Hospital	Physiotherapist	Smartinhaler + iRobi robot	Not specified	- Maintenance medication use	4 months	Mixed	- Patient: robot device - HCP: web browser - Patient + HCP: phone call interventions	- Patient: Robot programmed to deliver COPD management with several features: (1) measure of health variables; (2) oral and inhaled medication reminders and record of their adherence; (3) reminders of rehabilitation and related videos; (4) education about COPD with videos and pop-up messages; (5) Option of "I am feeling unwell" function on demand; and (6) show trends over time of health status and adherence to the patient. - HCP: access to data on "alert function" and parameters outside the normal range to develop interventions.
Chen (2019) [28]	COPD	Medical Clinic	Not specified (providers)	Propeller Health^®^	pMDI	- SABA use	12 months	Mixed	- Patient: Smartphone application or web platform - HCP: Web dashboard	- HCP: access to data from inhaler use to develop. interventions (support calls). - HCP + patients: Collected data could be used by providers to perform medication adjustments or early intervention at the sign of increasing SABA use. Fruthermore, at-risk notifications were sent to providers if a patient was considered at increased risk for an exacerbation (excessive SABA use). Appropriate follow-up via telephone or an in-person visit could be determined by providers. A respiratory therapist assisted patients with technical questions by phone and in person. - Patients: access to a web or mobile platform promoting self-management with information about their SABA use trends, environmental triggers and guidelines-based education.
Gregoriano (2019) [55]	Asthma, COPD	Hospital	Pharmacist Nurse	Depending on the inhaler: - Smartinhaler - POEMS	pMDI DPI	- SABA use - Maintenance medication use	6 months	Mixed	- Patient: Smartphone application with audio-reminder or alarm-clock - HCP: Online access to data - Phone call sessions	- Patient: audio-reminder generated by an app (for Smartinhaler devices) or an alarm clock (for POEMS) that was directly transferred to the participants’ smartphones (reminder independent from passive monitoring). - HCP: access to data from inhaler use to develop interventions (support calls). - Patient + HCP: support calls when needed.
Kaye (2021) [44]	COPD	Not specified	Not specified	Propeller Health^®^	pMDI	- CAT score - SABA use - Maintenance therapy use (if sensor compatible)	6 months	Patient (potentially mixed if the patients allowed access to their data)	Patient: Smartphone application	- Patient: self-management with evidence-based education, feedback on medication use and reminders for scheduled medications. [Patients had the option to share their information with their providers but were not required to do so.]
Kaye (2021) [49]	Asthma, COPD	Social media campaigns	Not specified	Propeller Health^®^	Not specified	- Maintenance medication use - ACT, CAT	8–97 Days	Patient	Patient: Smartphone application	- Patient: The app engaged the patient through evidence-based asthma and/or COPD content, feedback on medication use and trends, and schedule-based medication reminders through the sensor and smartphone application. In case of continued poor medication adherence, additional gamified features and challenges were presented to improve daily medication adherence. The app also prompts users to complete ACT and CAT test monthly.
Merchant (2016) [23]	Asthma	Hospital	Physician	Propeller Health^®^	pMDI	- SABA use - - ACT	12 meses	Mixed	- Patient: smartphone application - HCP: computer dashboard	- Patient: Self-management including data-driven asthma assessment and guidance (education, reminders and alerts) with the smartphone application. - HCP: access to dashboards and possibility to intervene with patients.
Moore (2021) [51]	Asthma	Hospital	Not specified	Propeller Health^®^	pMDI DPI	- SABA use - Maintenance medication use	6 months	Mixed	- Patient: smartphone application - HCP: computer dashboard - HCP + patient: email, phone, face-to-face meeting	- Patient: data on inhaler use, identification of potential triggers, etc. available in the smartphone application to improve self-management. - HCP: information of inhaler use in the computer dashboard. If necessary, the HCP could e-mail or phone the patient, or see them in the clinic to have an open, non-judgmental discussion.
Mosnaim (2021) [27]	Asthma	NorthShore University HealthSystem	Nurse (supervised by an allergist or immunologist)	Propeller Health^®^	Not specified	- SABA use - Maintenance ICS use	3 months	Mixed	- Patient: smartphone application - HCP: computer dashboard - HCP + patient: monthly phone calls	- Patient: self-management including access to ICS and SABA usage in the smartphone application and reminders for missed or late doses. - HCP: Access to dashboards and the possibility to intervene with patients if poor adherence was noted. In addition, monthly phone calls to provide feedback on ICS adherence and SABA use.
Nides (1993) [50]	COPD	Universities	Health educator	Nebulizer Chronolog	pMDI	- Maintenance medication use	4 months	Mixed	- HCP + patient: face-to-face review with the health educator and printed copies regarding their inhalers use.	- Patient + HCP: Participants in the feedback group had face-to-face reviews with the health educator and printed copies regarding their inhalers use. Praise was given for the areas in which usage was satisfactory, and individual behavioural strategies were collaboratively developed to address problem areas.
O’Dwyer (2020) [58]	Asthma, COPD	Community pharmacy	Pharmacist	INCA^TM^	DPI	- Maintenance salmeterol/fluticasone therapy use	6 months	HCP	- HCP: Online access to data - HCP + patient: face-to-face training sessions	- HCP: access to data on inhaler use to provide personalized visual feedback (time and technique of inhaler use) obtained by analysis of the data recorded to the INCA device and generation of visual graphs, which the pharmacist reviewed and discussed with the patient. - Patient + HCP: discussion of visual graphs
Onyirimba (2003) [61]	Asthma	Hospital	Pulmonologist Nurse	Chronologs	pMDI	- SABA use - Maintenance ICS use	10 weeks	HCP	- HCP: Access to computer printout data - Patient + HCP: face-to-face sessions	- HCP: access to data on inhaler use to provide personalized intervention in follow-up sessions when needed.
Sloots (2021) [45]	COPD	Hospital	Nurse (training)	Respiro^®^	DPI	- Maintenance therapy use - Inhalation technique - Daily symptoms - Health-related quality of life (SGRQ) - COPD self-management behaviour and knowledge (Partners in Health questionnaire) - COPD self-efficacy (COPD SelfEfficacy Score) - Anxiety/and depression score (HADS).	4 months	Patient	Patient: e-health application modules (tablet or PC) [Training: (a) Group session; (b) Phone calls]	- Patient: self-management intervention in which self-report of symptoms in diaries and data collected with an inhaler sensor were linked to an automated decision support system with actions communicated with an e-health application.
Sulaiman (2018) [53]	Asthma	Hospital	Nurse	INCA^TM^	DPI	- Maintenance salmeterol/fluticasone therapy use - PEF	3 months	HCP	- HCP: access to data - Patient + HCP: face-to-face sessions	- HCP: access to data from inhaler use to develop interventions. - Patient + HCP: interventional sessions guided by visual (bio)feedback on their adherence behaviours from information recorded with the sensor.
Van Sickle (2013) [47]	Asthma	Medical clinics and community care	Not specified	Not specified	pMDI	- Time, location, and use of SABA	4 months	Patient	- Patient: email reports - Patient: online interface	- Patients received weekly e-mail reports and had access to an online interface displaying maps and charts of their inhaler use, assessment of their asthma control and simple advice derived from the NAEPP guidelines.
Weinstein (2019) [62]	Asthma	Hospital	Allergist, clinical immunologist, pulmonologist	SmartTrack device	pMDI	- SABA use - Maintenance ICS/LABA use	3 months	Mixed	- Patient: web application - HCP: web application - Patient + HCP: motivational interviewing sessions	- HCP: Specific training to implement motivational interviewing adherence strategies + monitoring on data adherence + interventions based on a decision-making tool. The tool identified barriers and provided material for the interventions. - Written information and videos to reinforce the written response.
Yawn (2021) [48]	COPD	Inclusion based on an online registry of COPD patients	Not specified	Propeller Health^®^	pMDI DPI (Ellipta^®^ inhaler; GlaxoSmithKline BV, UK.	- SABA or SABA/SAMA use - Maintenance medication use	24 weeks	Patient	Patient: smartphone application	- The smartphone application allowed patients to check their medication adherence and inhaler use. Notifications were sent if their maintenance inhaler was not used for more than 4 days or in case of an increased rescue therapy use. Participants also received maintenance medication reminders according to their schedule. Participants could also receive feedback on rescue mediation use and had access to evidence-based COPD-related information on triggers and symptoms.

## Data Availability

Data sharing not applicable.

## Supplementary Materials

The following supporting information can be downloaded at: https://www.mdpi.com/article/10.3390/ph16030414/s1, File S1: Supplement 1. Search strategy; File S2: Supplement 2. Variables assessed in clinical trials.
