# Supplementary material for: Clinical Impact of Electronic Monitoring Devices of Inhalers in Adults with Asthma or COPD: A Systematic Review and Meta-Analysis
Source: Pharmaceuticals (Basel). 2023 Mar 8;16(3):414. doi: 10.3390/ph16030414 (PMC10055893; doi:10.3390/ph16030414)
Supplement: Supplementary file 1 [file pharmaceuticals-16-00414-s001.zip › PRESS 2015 Guideline Evidence-Based Checklist.pdf]

PRESS 2015 Guideline Evidence-Based Checklist

1. Translation of the research question

- **Does the search strategy match the research question/PICO?**
  - The authors agree that the topic assessed in this review (clinical impact of electronic monitoring devices used in inhalers) is a very new approach with recent evidence that needs to be reviewed and a meta-analysis (if possible) to gain more knowledge. The PICO question totally matches the research question, as we focused on adults with asthma/COPD using these devices with the aim to get better health outcomes compared with other interventions or the standard of care.
- **Are the search concepts clear?**
  - We identified the four domains of PICO: P (asthma and COPD, adults), I (use of electronic monitoring devices of inhalers), C (any comparator, as justified in the text); O (clinical impact – several variables related to management of the respiratory disease included)
- **Are there too many or too few PICO elements included?**
  - All elements are included
- **Are the search concepts too narrow or too broad?**
  - The concepts are appropriate for the search. This issue was approached and handled in an exploratory phase (searching terminology in reference articles previously to gain a broader perspective to construct the search).
- **Does the search retrieve too many or too few records? (Please show number of hits per line.)**
  - As we are assessing a very innovative monitoring approach with the corresponding interventions, terminology can vary greatly depending on the study. Thus, we had to include a range of keywords that resulted in a relatively great number of results, but it is justified, as also commented in the text and PRISMA flow diagram.
- **Are unconventional or complex strategies explained?**
  - There are no unconventional/complex strategies in this review.

2. Boolean and proximity operators (these vary based on search service)

- **Are Boolean or proximity operators used correctly?**
  - The authors used clear and simple Boolean operators, correctly.
- **Is the use of nesting with brackets appropriate and effective for the search?**
  - The authors used brackets appropriately and effectively to perform the search.
- **If NOT is used, is this likely to result in any unintended exclusions?**
  - Not applicable.
- **Could precision be improved by using proximity operators (eg, adjacent, near, within) or phrase searching instead of AND?**
  - The authors did not used proximity operators in the review but the search implied a previous task with research articles focusing on the topic to detect the terminology used in this type of papers, so that we obtained similar or

synonym phrases that allowed us to keep a simpler Boolean approach. Also, the results of the search in the databases gave us a great number of results, that would not be significantly different compared to have used other operators. Also, we used an additional search in webpages of the devices with related articles, and revising cites of articles, etc. so that we ensured an optimized search.

- **Is the width of proximity operators suitable (eg, might adj5 pick up more variants than adj2)?**
  - Not applicable.

3. Subject headings (database specific)

- **Are the subject headings relevant?**
  - We did not used subject heading deliberately since we were covering a topic which is a very novel approach (monitoring devices in inhalers with associated interventions) that not match any heading. This is why we used free text searching with keywords that have been carefully selected according to a previous effort of researching articles on the topic to detect and list potential keywords of interest. Also, we decided to cover asthma and COPD with free text search.
- **Are any relevant subject headings missing; for example, previous index terms?**
  - Not applicable
- **Are any subject headings too broad or too narrow?**
  - Not applicable
- **Are subject headings exploded where necessary and vice versa?**
  - Not applicable
- **Are major headings (''starring'' or restrict to focus) used? If so, is there adequate justification?**
  - Not applicable
- **Are subheadings missing?**
  - Not applicable
- **Are subheadings attached to subject headings? (Floating subheadings may be preferred.)**
  - Not applicable
- **Are floating subheadings relevant and used appropriately?**
  - Not applicable
- **Are both subject headings and terms in free text (see the following) used for each concept?**
  - Not applicable

4. Text word searching (free text)

- **Does the search include all spelling variants in free text (eg, UK vs. US spelling)?**
  - No variants were found in this case.
- **Does the search include all synonyms or antonyms (eg, opposites)?**
  - As aforementioned, we performed a previous exploratory search with articles, references and their cites to detect the domains of interest and their corresponding terminology and synonyms, as explained in the text.
- **Does the search capture relevant truncation (ie, is truncation at the correct place)?**
  - We used relevant truncation to obtain as much output of interest for the review as possible.
- **Is the truncation too broad or too narrow?**
  - The truncation was considered adequate for the review. Considering the novelty of the systems and interventions assessed, we preferred to design a search strategy that may result in a broad result rather than losing articles of interest (although it may have resulted in a higher number of initial results to assess by the research team).
- **Are acronyms or abbreviations used appropriately? Do they capture irrelevant material? Are the full terms also included?**
  - We almost did not used abbreviations unless universally used and accepted (COPD). For the rest of keywords, no clear acronyms were detected.
- **Are the keywords specific enough or too broad? Are too many or too few keywords used? Are stop words used?**
  - As previously stated, we conducted an initial exploratory search phase to detect terminology used in this type of studies, as this is a very recent clinical approach. This information is extensively explained in the text. We used a relatively great number of keywords as there is variability in the terms used for monitoring of inhalers because a restrictive list would results in losing articles of interest.
- **Have the appropriate fields been searched; for example, is the choice of the text word fields (.tw.) or all fields (.af.) appropriate? Are there any other fields to be included or excluded (database specific)?**
  - We selected filters in each database: title, abstract, keywords; except for some databases in which only title, abstract was only available. See text of the review.
- **Should any long strings be broken into several shorter search statements?**
  - This issue was considered adequate.

5. Spelling, syntax, and line numbers

- **Are there any spelling errors?**
  - No spelling errors were detected.
- **Are there any errors in system syntax; for example, the use of a truncation symbol from a different search interface?**
  - No errors in system syntax were detected.
- **Are there incorrect line combinations or orphan lines (ie, lines that are not referred to in the final summation that could indicate an error in an AND or OR statement)?**

- No errors in this regard were detected.

6. Limits and filters

- **Are all limits and filters used appropriately and are they relevant given the research question?**
  - All limits and filters used were considerate appropriate. We did not use limits for time. We did not use limits for language (exclusions were performed later by reviewers). As aforementioned, we used filters for title, abstract and keywords.
- **Are all limits and filters used appropriately and are they relevant for the database?**
  - Limits and filters were used appropriately.
- **Are any potentially helpful limits or filters missing? Are the limits or filters too broad or too narrow? Can any limits or filters be added or taken away?**
  - No helpful limits or filters are missing.
- **Are sources cited for the filters used?**
  - Not applicable.
