# Supplementary material for: Clinical Impact of Electronic Monitoring Devices of Inhalers in Adults with Asthma or COPD: A Systematic Review and Meta-Analysis
Source: Pharmaceuticals (Basel). 2023 Mar 8;16(3):414. doi: 10.3390/ph16030414 (PMC10055893; doi:10.3390/ph16030414)
Supplement: Supplementary file 1 [file pharmaceuticals-16-00414-s001.zip › PRISMA_checklist.pdf]

PRISMA 2020 Checklist

Section and Topic	Item #	Checklist item	Location where item is reported
**TITLE**			
Title	1	Identify the report as a systematic review.	Item reported in the title
**ABSTRACT**			
Abstract	2	See the PRISMA 2020 for Abstracts checklist.	Item reported in the abstract section. PRISMA guidelines for abstracts were followed, adapted to the requirements of the journal
**INTRODUCTION**			
Rationale	3	Describe the rationale for the review in the context of existing knowledge.	Rationale for the review is provided throughout the introduction section. The introduction covers the relevance of asthma and COPD, the importance and problems of adherence to inhalers, the problems of adherence assessment, the novel approach of EMDs, the possibilities of EMDs and the lack of evidence of their use on clinical outcomes.
Objectives	4	Provide an explicit statement of the objective(s) or question(s) the review addresses.	An explicit statement of the objectives is provided in the last paragraph of the introduction section.
**METHODS**			
Eligibility criteria	5	Specify the inclusion and exclusion criteria for the review and how studies were grouped for the syntheses.	Included in the “Methods” section, “Eligibility criteria” subsection.
Information sources	6	Specify all databases, registers, websites, organisations, reference lists and other sources searched or consulted to identify studies. Specify the date when each source was last searched or consulted.	Included in the “Methods” section, “Information sources” subsection.
Search strategy	7	Present the full search strategies for all databases, registers and websites, including any filters and limits used.	Included in the “Methods” section, “search strategy” subsection.
Selection process	8	Specify the methods used to decide whether a study met the inclusion criteria of the review, including how many reviewers screened each record and each report retrieved, whether they worked independently, and if applicable, details of automation tools used in the process.	Included in the “Methods” section, “Selection and Data Collection process” subsection.
Data collection process	9	Specify the methods used to collect data from reports, including how many reviewers collected data from each report, whether they worked independently, any processes for obtaining or confirming data from study investigators, and if applicable, details of automation tools used in the process.	Included in the “Methods” section, “Selection and Data Collection process” subsection.
Data items	10a	List and define all outcomes for which data were sought. Specify whether all results that were compatible with each outcome domain in each study were sought (e.g. for all measures, time points, analyses), and if not, the methods used to decide which results to collect.	Included in the “Methods” section, “Data items and synthesis methodss” subsection.
	10b	List and define all other variables for which data were sought (e.g. participant and intervention characteristics, funding sources). Describe any assumptions made about any missing or unclear information.	Included in the “Methods” section, “Data items and synthesis methodss” subsection.
Study risk of bias assessment	11	Specify the methods used to assess risk of bias in the included studies, including details of the tool(s) used, how many reviewers assessed each study and whether they worked independently, and if applicable, details of automation tools used in the process.	Included in the “Methods” section, “Study and report of risk of bias assessment” subsection.
Effect measures	12	Specify for each outcome the effect measure(s) (e.g. risk ratio, mean difference) used in the synthesis or	Included in the “Methods” section, “Statistical

Section and Topic	Item #	Checklist item	Location where item is reported
		presentation of results.	analysis" subsection.
Synthesis methods	13a	Describe the processes used to decide which studies were eligible for each synthesis (e.g. tabulating the study intervention characteristics and comparing against the planned groups for each synthesis (item #5)).	Included in the "Methods" section, "Statistical analysis" subsection + "Eligibility criteria" subsection.
	13b	Describe any methods required to prepare the data for presentation or synthesis, such as handling of missing summary statistics, or data conversions.	Included in the "Methods" section, "Statistical analysis" subsection.
	13c	Describe any methods used to tabulate or visually display results of individual studies and syntheses.	Included in the "Methods" section, "Statistical analysis" subsection.
	13d	Describe any methods used to synthesize results and provide a rationale for the choice(s). If meta-analysis was performed, describe the model(s), method(s) to identify the presence and extent of statistical heterogeneity, and software package(s) used.	Included in the "Methods" section, "Statistical analysis" subsection.
	13e	Describe any methods used to explore possible causes of heterogeneity among study results (e.g. subgroup analysis, meta-regression).	Included in the "Methods" section, "Statistical analysis" subsection.
	13f	Describe any sensitivity analyses conducted to assess robustness of the synthesized results.	Included in the "Methods" section, "Statistical analysis" subsection.
Reporting bias assessment	14	Describe any methods used to assess risk of bias due to missing results in a synthesis (arising from reporting biases).	Included in the "Methods" section, "Statistical analysis" subsection.
Certainty assessment	15	Describe any methods used to assess certainty (or confidence) in the body of evidence for an outcome.	Included in the "Methods" section, "Statistical analysis" subsection.
**RESULTS**			
Study selection	16a	Describe the results of the search and selection process, from the number of records identified in the search to the number of studies included in the review, ideally using a flow diagram.	Figure 2 includes a flow diagram with the data requested. Also, information is provided in the "Methods" section, "Statistical analysis" subsection.
	16b	Cite studies that might appear to meet the inclusion criteria, but which were excluded, and explain why they were excluded.	Included in the "Results" section, "Study selection" subsection.
Study characteristics	17	Cite each included study and present its characteristics.	Included in table 1, table 2 and the "Results" section, "Study characteristics and results" subsection.
Risk of bias in studies	18	Present assessments of risk of bias for each included study.	Included in the "Results" section, "Risk of bias in studies" subsection and in Figure 3
Results of individual studies	19	For all outcomes, present, for each study: (a) summary statistics for each group (where appropriate) and (b) an effect estimate and its precision (e.g. confidence/credible interval), ideally using structured tables or plots.	Included in Table 1 and the "Results" section, "Study characteristics and results
Results of syntheses	20a	For each synthesis, briefly summarise the characteristics and risk of bias among contributing studies.	Included in the "Results" section, "Risk of bias in studies" subsection and partially in the subsection "Impact of interventions on adherence to maintenance inhaled therapy"
	20b	Present results of all statistical syntheses conducted. If meta-analysis was done, present for each the summary estimate and its precision (e.g. confidence/credible interval) and measures of statistical heterogeneity. If comparing groups, describe the direction of the effect.	Included in the "Results" section, subsections:<br>- Impact of interventions on adherence to maintenance inhaled therapy

PRISMA 2020 Checklist

Section and Topic	Item #	Checklist item	Location where item is reported
			- Impact of interventions on other clinical outcomes
	20c	Present results of all investigations of possible causes of heterogeneity among study results.	We present differences across studies that could impact at this level (methodology used, HCPs involved, types of interaction, interventions, etc.) in Table 2.
	20d	Present results of all sensitivity analyses conducted to assess the robustness of the synthesized results.	We present results of adherence in studies >3 months duration. Also, we present in <3 months which shows similar results (although these lasta analysis onlys shows a tendency, the results are not statistically significant). We performed additional analyses with all results, with positive results.
Reporting biases	21	Present assessments of risk of bias due to missing results (arising from reporting biases) for each synthesis assessed.	Included in the "Results" section, "Risk of bias in studies" subsection and in Figure 3
Certainty of evidence	22	Present assessments of certainty (or confidence) in the body of evidence for each outcome assessed.	Results section
**DISCUSSION**			
Discussion	23a	Provide a general interpretation of the results in the context of other evidence.	We present this information in paragraphs 1 to 5 of the discussion section.
	23b	Discuss any limitations of the evidence included in the review.	We present this information in the sixth paragraph of the discussion section.
	23c	Discuss any limitations of the review processes used.	We present this information in the sixth paragraph of the discussion section.
	23d	Discuss implications of the results for practice, policy, and future research.	We present this information in several paragraphs of the discussion, and especially in the last one.
**OTHER INFORMATION**			
Registration and protocol	24a	Provide registration information for the review, including register name and registration number, or state that the review was not registered.	Included in the "Methods" section, "Study registration" subsection
	24b	Indicate where the review protocol can be accessed, or state that a protocol was not prepared.	Included in the "Methods" section, "Study registration" subsection. The protocol can be accessed with the title or the identification code.
	24c	Describe and explain any amendments to information provided at registration or in the protocol.	-
Support	25	Describe sources of financial or non-financial support for the review, and the role of the funders or sponsors in the review.	Non-financial support in the review.
Competing interests	26	Declare any competing interests of review authors.	Competing of interest was declared to the journal.
Availability of data, code and	27	Report which of the following are publicly available and where they can be found: template data collection forms; data extracted from included studies; data used for all analyses; analytic code; any other materials used	-Data extracted from included studies

Section and Topic	Item #	Checklist item	Location where item is reported
other materials		in the review.	- Data used for analyses<br>- Supplement 1<br>- Supplement 2: variables assessed in clinical trials

From: Page MJ, McKenzie JE, Bossuyt PM, Boutron I, Hoffmann TC, Mulrow CD, et al. The PRISMA 2020 statement: an updated guideline for reporting systematic reviews. BMJ 2021;372:n71. doi: 10.1136/bmj.n71

For more information, visit: http://www.prisma-statement.org/
