# Supplementary material for: Clinical Impact of Electronic Monitoring Devices of Inhalers in Adults with Asthma or COPD: A Systematic Review and Meta-Analysis
Source: Pharmaceuticals (Basel). 2023 Mar 8;16(3):414. doi: 10.3390/ph16030414 (PMC10055893; doi:10.3390/ph16030414)
Supplement: Supplementary file 1 [file pharmaceuticals-16-00414-s001.zip › Supplement 1.pdf]

# SUPPLEMENT 1. SEARCH STRATEGY

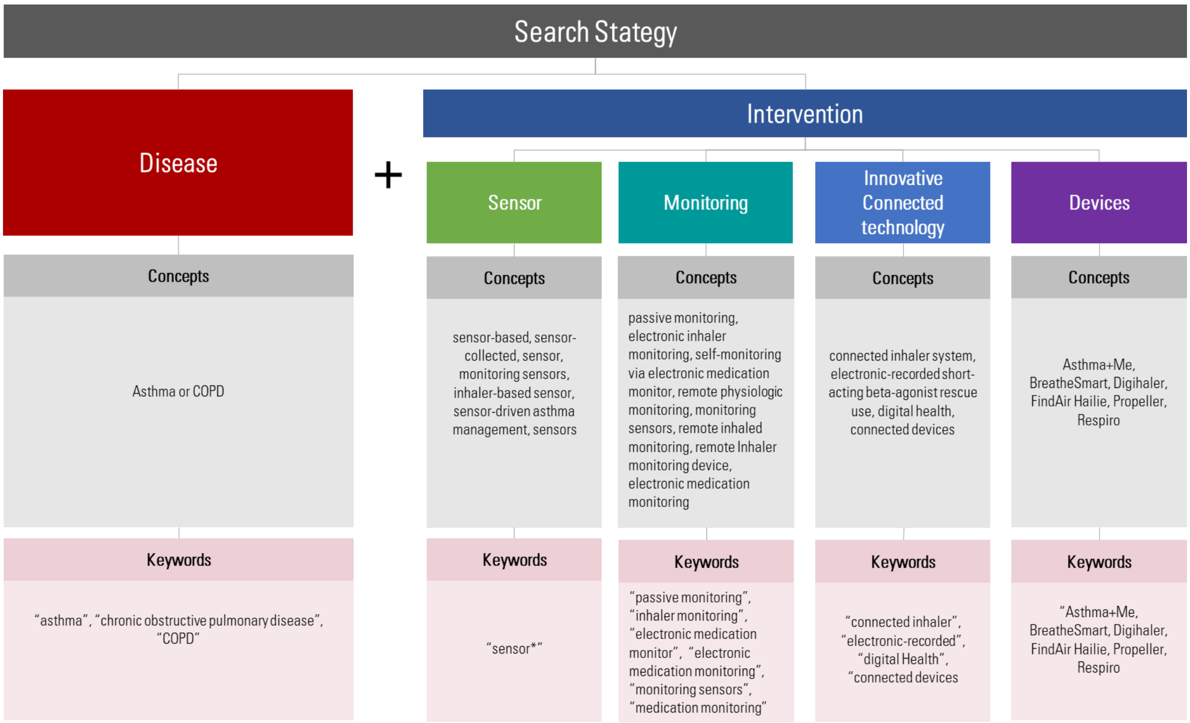


Figure. Search strategy summary

## 1. PUBMED search (filter: abstract/title):

(asthma or copd or "chronic obstructive pulmonary disease")

AND

(sensor* OR "passive monitoring" OR "inhaler monitoring" OR "electronic medication monitor" OR "electronic medication monitoring" OR "monitoring sensors" OR "medication monitoring" OR "Connected inhaler" OR "electronic-recorded" OR "digital Health" OR "Connected devices" OR "Asthma+Me" OR "BreatheSmart" OR "Digihaler" OR "FindAir Hailie" OR "Propeller" OR "respiro")

## 2. EMBASE search (filter: abstract/title/keywords):

(asthma or copd or "chronic obstructive pulmonary disease")

AND

(sensor* OR "passive monitoring" OR "inhaler monitoring" OR "electronic medication monitor" OR "electronic medication monitoring" OR "monitoring sensors" OR "medication monitoring" OR "Connected inhaler" OR "electronic-recorded" OR "digital Health" OR "Connected devices" OR "Asthma+Me" OR "BreatheSmart" OR "Digihaler" OR "FindAir Hailie" OR "Propeller" OR "respiro")

## 3. WEB OF SCIENCE search (filter: abstract/title):

(asthma or copd or "chronic obstructive pulmonary disease")

AND

(sensor* OR "passive monitoring" OR "inhaler monitoring" OR "electronic medication monitor" OR "electronic medication monitoring" OR "monitoring sensors" OR "medication monitoring" OR "Connected inhaler" OR "electronic-recorded" OR "digital Health" OR "Connected devices" OR "Asthma+Me" OR "BreatheSmart" OR "Digihaler" OR "FindAir Hailie" OR "Propeller" OR "respiro")

## 4. SCOPUS search (filter: title/abstract/keywords):

(asthma or copd or "chronic obstructive pulmonary disease")

AND

(sensor* OR "passive monitoring" OR "inhaler monitoring" OR "electronic medication monitor" OR "electronic medication monitoring" OR "monitoring sensors" OR "medication monitoring" OR "Connected inhaler" OR "electronic-recorded" OR "digital Health" OR "Connected devices" OR "Asthma+Me" OR "BreatheSmart" OR "Digihaler" OR "FindAir Hailie" OR "Propeller" OR "respiro")

## 5. COCHRANE search (filter: title/abstract/keywords):

(asthma or copd or "chronic obstructive pulmonary disease")

AND

(sensor* OR "passive monitoring" OR "inhaler monitoring" OR "electronic medication monitor" OR "electronic medication monitoring" OR "monitoring sensors" OR "medication monitoring" OR "Connected inhaler" OR "electronic-recorded" OR "digital Health" OR "Connected devices" OR "Asthma+Me" OR "BreatheSmart" OR "Digihaler" OR "FindAir Hailie" OR "Propeller" OR "respiro")

## ADDITIONAL SEARCH

An additional search was performed including cites in reviews found in the previous search, citation examination of the selected articles and in the websites Digihaler® (www.digihalerhcp.com), HeroTracker® (www.coherohealth.com), Respiro Amiko® (www.amiko.io), CapMedic® (www.capmedicinhaler.com), Turbuplus™ (www.turbuplusinfo.co.uk), Turbuplus™ (www.turbuplusinfo.co.uk), Propeller Health® (www.propellerhealth.com), INCA™ (www.incadevice.com), Hailie® (www.hailie.com)
