# Supplementary material for: Clinical Impact of Electronic Monitoring Devices of Inhalers in Adults with Asthma or COPD: A Systematic Review and Meta-Analysis
Source: Pharmaceuticals (Basel). 2023 Mar 8;16(3):414. doi: 10.3390/ph16030414 (PMC10055893; doi:10.3390/ph16030414)
Supplement: Supplementary file 1 [file pharmaceuticals-16-00414-s001.zip › supplement 2.pdf]

SUPPLEMENT 2: Variables assessed in clinical trials

DOMAIN	ASSESSED OUTCOMES
Adherence	- %: Moore (4-6months), Sulaiman (3 months), O'Dwyer (6 months), Broadbent (4 months), Onyirimba (10 weeks, no numerical data of the results; graphical data available)
	- Difference in mean ICS adherence (%) from baseline to the end of the study: Mosnaim (3 months period, they include data on baseline, 3 months and difference), Apter (6 months period, they include data on baseline and 6 months)
	- % of days in target range of adherence for puff inhalers (80-100%): Gregoriano (6 months)
	- % of days in target range of adherence for dry powder (80-100%): Gregoriano (6 months)
	- Average adherence from dose counter: Sulaiman (3 months)
	- Attempted adherence rate: Sulaiman (3 months), O'Dwyer (6 months = intentional initiation of medication use at the correct time)
	- Overdoses: Sulaiman (3 months)
	- Missed doses: Sulaiman (3 months)
	- Technique error rate: Sulaiman (3 months)
	- Adherence - Timing adherence for puff inhalers (proportion of correct dosing intervals during 24 h): Gregoriano (6 months)
	- Adherence - Timing adherence for dry powder (proportion of correct dosing intervals during 24 h): Gregoriano (6 months)
	- Adherence - Gaps for puff inhalers (proportion of days without inhalation): Gregoriano (6 months)
	- Adherence - Gaps for inhalation with dry powder capsules (proportion of days without inhalation): Gregoriano (6 months)
	- Adherence - Maximal gap length for puff inhalers: Gregoriano (6 months)
	- Adherence - Maximal gap length for dry powder capsules: Gregoriano (6 months)
	- Adherence - Proportion with actual adherence 80%: O'Dwyer (6 months)
	- Adherence - Proportion with actual adherence <50%: O'Dwyer (6 months)
	- Adherence - Critical technique error rate: O'Dwyer (over 6 months)
Rescue Medication ("SABA use")	- Mean monthly % rescue free-days (6 months): Moore (comparison between groups),
	- SABA free days %: Merchant (analysis of difference from baseline to 12 months, data of each time in % is available), Mosnaim (from run in period to final period of 3 months, they include data on baseline and 3 months)
	- SABA use: Merchant (Mean daily SABA use, analysis of difference from baseline to 12 months, data of each time in % is available), Onyirimba (10 weeks 24-hour and nigh time)
	- Mean total rescue medication use (Moore: baseline, 4-6 months)

Symptoms Control	- ACT score: Moore (4-6 months)
	- ACT controlled (% patients >19): Merchant (analysis in the change from baseline between the groups; baseline to 12 months)
	- ACT score difference from baseline: Merchant
	- 10% increase in SABA-free days: Mosnaim (3 month)
	- ACT score improvement >3 points: Mosnaim (3 month)
	- ACT score from <20 to >20: Mosnaim (3 month)
	- ACQ: Apter (change from baseline to 6 months, data available on baseline and end of the study), Weinstein (data at baseline and 3 months)
	- Asthma control improvement (composite variable): Mosnaim (3 month)
	- Clinical outcomes (composite variable including adherence, PEF, AQLQ, exacerbations): Sulaiman
FEV1	- FEV1 (difference from baseline): Apter (change of % from baseline to 6 months, data available on baseline and end of the study), Onyirimba (10 weeks, only difference from baseline), Weinstein (3 months in %)
Exacerbations	- Patients with at least 1 exacerbation: Mosnaim (3 months),
	- Frequency of total exacerbations during the study period: Gregoriano (6 months), O'Dwyer (6 month)
	- Time to next exacerbation: Gregoriano (data of the 6-month period)
	- ≥1 course of oral systemic corticosteroids: Mosnaim (3 months)
	- Hospitalizations or ED visits: Mosnaim (3 months)
	- ED visits for asthma: Apter (12 months, change from baseline analysis, they have data on baseline and 6 months)
	- ED visits for any cause: Apter (12 months, change from baseline analysis, they have data on baseline and 6 months)
	- Hospitalization for asthma: Apter (12 months, change from baseline analysis, they have data on baseline and 6 months)
	- Hospitalization for any cause: Apter (12 months, change from baseline analysis, they have data on baseline and 6 months)
	- Number of hospitalizations: Broadbent (4 months)
	- Number of days in hospital for respiratory problems: Broadbent (4 months)
Quality of life	- Quality of life (mAQLQ; difference from baseline): Apter (AQLS, difference from baseline to 6 months, data on baseline and end of the study available), Onyirimba (10 weeks, only information of the increase)
	- QOL (SGRQ): Gregoriano (6 months change, data available of baseline and change after 6 months), O'Dwyer (6 months change, information only of difference from baseline and only in one of the 3 groups, other groups without information)
	- Self-reported symptoms (6 months): a) cough; b) breathlessness; c) nocturnal symptoms; d) wheeze: O'Dwyer (6 months)
	- CCQ total (difference from baseline): Broadbent (4 months, data on baseline, 4 months and difference)

NOTE: 1. For some studies, data on several times was provided. In that case, the longer period is presented in this table; 2. In some cases, results regarding a specific variable were also reported alternatively in descriptive analysis (e.g. “descriptive analysis”: % adherence vs “assessed outcome” difference in adherence). Data on the table refers to the outcomes assessed in studies.
